# High‐resolution micro‐CT scanning as an innovative tool for evaluating dental hard tissue development

**DOI:** 10.1120/jacmp.v15i4.4956

**Published:** 2014-07-08

**Authors:** Guangyan Dong, Qianqian Dong, Yi Liu, Beiyan Lou, Jin Feng, Kejing Wang, Xuedong Zhou, Hongkun Wu

**Affiliations:** ^1^ State Key Laboratory of Oral Diseases West China College of Stomatology, Sichuan University Chengdu China; ^2^ Department of Endodontics Hospital of Stomatology Xi'an Jiaotong University Xi'an 710049 China; ^3^ Department of Oral Medicine Sichuan Academy of Medical Sciences & Sichuan Provincial People's Hospital Chengdu 610065 China; ^4^ Department of Geriatric Diagnosis West China College of Stomatology Sichuan University Chengdu 610065 China

**Keywords:** dental hard tissue development, microcomputerized tomography, three‐dimensional reconstruction

## Abstract

Microcomputerized tomography (micro‐CT) allows discriminating very small changes in dental hard tissue volumes. The aim of the present study was to create a new method for obtaining high‐resolution, three‐dimensional images of dental hard tissue development using micro‐CT, and to observe the changes in dental hard tissue development and composition in growing rat pups. Tooth germs from rats at the end of the 20‐day embryonic period (E20) and during the neonatal period (D1‐14) were subjected to micro‐CT. Three‐dimensional reconstructions were analyzed to compare dental hard tissue formation and mineralization during the different development periods. Scanning electron microscopy and energy dispersive spectroscopy were used to confirm mineral density (MD). Dental hard tissue began to form during the E20, but the process was slow and resulted in minimal deposition. Hard tissue volume increased by approximately 0.040 mm^3^/day from E20 to D3, and by 0.12‐0.42 mm^3^/day after D3, peaking at 0.42 mm^3^/day at D12. This increase in hard tissue volume resulted in continuous increases in hard tissue thickness, from 90.0 ± 20.7 μm at E20 to 545.2 ± 14.1 μm by D14. MD was 298 ± 3.1 mg HA/cm at E20 and increased to 678.2 ± 6.1 mg HA/cm by D14. The degree of calcification also progressively increased during the first 14 days of development. Dental MD was strongly associated with calcification. This study indicates that micro‐CT is a nondestructive, high‐resolution, reliable, and innovative tool for the evaluation of volume and MD of dental hard tissues during development. Micro‐CT minimizes artifacts caused by sample preparation.

PACS number: 87

## I. INTRODUCTION

Dental hard tissue development is a highly orchestrated dynamic process, culminating in the formation of complex mineralized structures that are optimized for specific functions. Accurate and efficient three‐dimensional (3D) image can help to identify the progressive changes in 3D dental hard tissue morphology and density occurring during postnatal growth. Previous researches used conventional radiography, conventional computerized tomography (CT), or histological preparations to study dental hard tissue development.^1^, ^2^, ^3^ In these studies, images were obtained by physical sectioning, greatly compromising precision. Traditionally, mineral concentration distribution and density in dental hard tissue were measured using both direct (chemical analysis of a microsample) and indirect methods (contact microradiography).^(4)^ However, these techniques are sample‐destructive, as well as time‐consuming.

X‐ray micro‐CT is increasingly used in dental research to provide accurate and detailed images of the morphological characteristics of teeth, without any tooth sample destruction.^1^, ^2^, ^3^, ^5^, ^6^, ^7^ Using this technique, images collected from each plane can be reconstructed to produce 3D density maps.^2^, ^8^, ^9^ Micro‐CT systems may be used to measure the mineral concentration of teeth with a high accuracy (variation coefficient of <1%), and at a resolution of 5−30 μm,^10^, ^11^, ^12^, ^13^ and to indirectly assess mineral density (MD).^14^, ^15^, ^16^ Micro‐CT may also be used in a clinical setting.^17^, ^18^, ^19^, ^20^, ^21^ and in archeology.^1^, ^22^, ^23^, ^24^, ^25^

To the best of our knowledge, 3D imaging of dental hard tissue development and quantitative MD evaluation were not previously investigated using micro‐CT. The present study was performed to determine whether micro‐CT was sensitive enough to evaluate 3D image data of dental hard tissue development without the need for specimen preparation and destruction. We also assessed how closely 3D image data reflected the detailed morphological changes in dental hard tissue occurring during development, and investigated whether quantitative analysis of MD accurately represented the degree of mineralization in developing dental tissue. This study will allow a precise description of the formation of hard dental tissue during development without artifacts due to sample preparation. This study suggests that micro‐CT is a promising method to monitor tooth germ development, and that it can be used for basic and clinical dental research.

## II. MATERIALS AND METHODS

### A. Animals

Forty healthy adult SD rats (30 females and 10 males) from the Experimental Animal Center of the Sichuan University (Chengdu, China) were maintained in a virus‐ and parasite‐free facility, and fed a standard diet. Females had no reproductive history. Vaginal suppositories were checked the morning after mating and embryonic day 0.5 (E0.5) was set at 12:00 h on the day when a positive vaginal plug was found.

The study was approved by the institutional review board and the Animal Care and Use Committee of the State Key Laboratory of Oral Diseases, Sichuan University (Chengdu, China).

### B. Dental hard tissue harvesting

**Figure 1:**
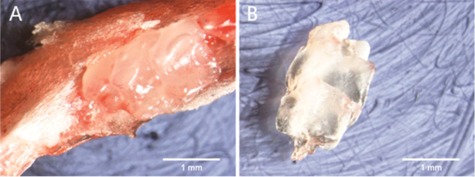
Removal of mandibular bone (a) covering the tooth germ to reveal the tooth germ. Complete tooth germ (b).

Pregnant females were sacrificed at E20 to obtain fetal rats (n=10). On days 1 to 14 (D1‐D14), ten pups were sacrificed each day. Mandibles were dissected from the soft tissues, and were fixed in 4% paraformaldehyde for 24 h. One side was used for micro‐CT, and the other was used for scanning electron microscopy (SEM). Corresponding molar tooth germs on both sides were removed using a stereomicroscope (Fig. 1).

### C. Micro‐CT

**Figure 2:**
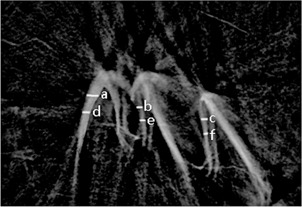
Hard tissue thickness was measured at points marked by a, b, c, d, e, and f.

We used a μCT80 micro‐CT system (SCANCO Medical, Bruttisellen, Switzerland). The analysis software was calibrated using a control disc made of hydroxyapatite (HA). Micro‐CT was set at a source voltage of 55 kV and a current of 170 mA. Each scan yielded an image data set of 1024×1024 2D axial slices acquired in ten μm sections. Twenty slices were selected to measure hard tissue thickness. On each slice, images from three cusp tips were divided into three equal portions, using six trisection points (marked a, b, c, d, e, and f) (Fig. 2). The average length at the six points represented the hard tissue thickness. Hard tissue volume and MD were simultaneously obtained from the analysis software.

### D. Scanning electron microscopy

Samples were cycled through distilled water, 70%, 90%, and 100% alcohol and xylenes, embedded in methyl methacrylate, washed in running water, rinsed several times in distilled water, and air‐dried at room temperature. Samples were mounted on SEM stubs. The examined surface was sputter‐coated with gold palladium, and viewed at an acceleration voltage of 15 kV using a SEM/EDS microscope system (Hitachi S3400; Hitachi Corp., Tokyo, Japan). Calcium and phosphorus levels at the cusp tip, at the edge of the root direction, and at the midpoint between the two, were analyzed using energy dispersive spectroscopy (EDS). The ratio of calcium atoms/calcium+phosphorus atoms (${\text{Ca}}^{2+}/P^{3-}+{\text{Ca}}^{2+}$) was used as a measure of calcification.

### E. Statistical analysis

Data were analyzed using SPSS 13.0 (SPSS Inc., Chicago, IL). Data are presented as means and standard deviations.

## III. RESULTS

### A. Morphological observation of dental hard tissue

**Figure 3:**
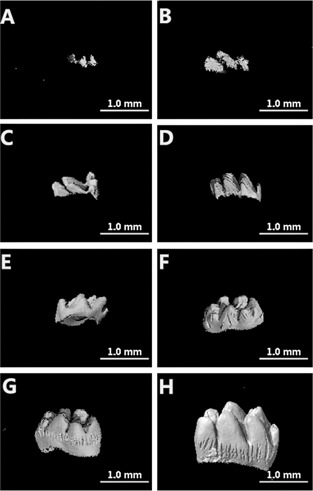
At E20, minerals and calcium were mainly deposited in the tooth cusp (a). At D1 (b), minerals and calcium in the tooth cusp increased. At D3 (c), minerals became integrated. Minerals were completely integrated by D5 (d). At D7 (e), tooth cusp elongated. At D10 (f), a complete crown had formed. At D12 (g), minerals deposited to the root. At D14 (h), tooth cusps were elongated, and minerals deposition to the root were increased.

Micro‐CT showed the typical developmental morphology of dental hard tissues. At E20, minerals and calcium deposits were mainly observed in the tooth cusp (Fig. 3(a)). At D1, minerals and calcium deposits in the tooth cusp were increased (Fig. 3(b)). At D3, the minerals became integrated (Fig. 3(c)). At D5, the minerals were completely integrated (Fig. 3(d)). At D7, the tooth cusp became elongated (Fig. 3(e)). At D10, a complete crown was formed (Fig. 3(f)). At D12, minerals deposited to the root (Fig. 3(g)). Finally, at D14, the tooth cusps were tall, and the minerals deposited to the root were increased (Fig. 3(h)).

### B. Morphological measurement of dental hard tissues

**Figure 4:**
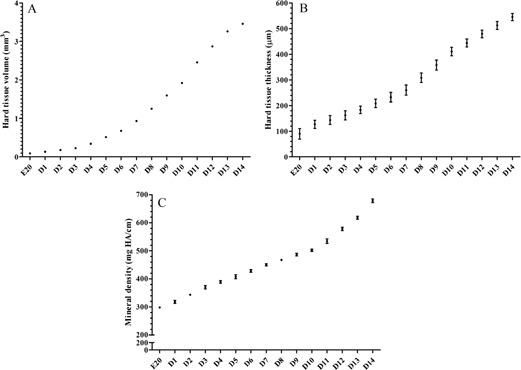
Changes in time (from E20 to D14) of (a) hard tissue volume, (b) hard tissue thickness, and (c) mineral density. N=10/time point. Results are presented as means ± SD.

Dental hard tissues began to form during the E20, but the process was slow and resulted in minimal deposition, with the volume of hard tissue increasing by approximately $0.040\;{\text{mm}}^{3}$ per day from E20 to D3, and the thickness increasing by approximately 30 μm per day during the same period. Thereafter, there was a progressive increase in hard tissue volume growth rate by $0.12‐0.42\;{\text{mm}}^{3}/\text{day}$ after D3, peaking at $0.42\;{\text{mm}}^{3}/\text{day}$ at D12 (Fig. 4).

### C. Changes in mineral density

Micro‐CT revealed gradual changes in dental hard tissue during various stages of embryogenesis (Fig. 4). At E20, MD was 298.0±3.1 mg HA/cm. At this time, dental hard tissue had just begun to form and the degree of calcification was relatively low. MD progressively increased during the first days of life, reaching a mean of 678.2±6.1 mg HA/cm by D14.

### D. Calcium and phosphorus determination

**Figure 5:**
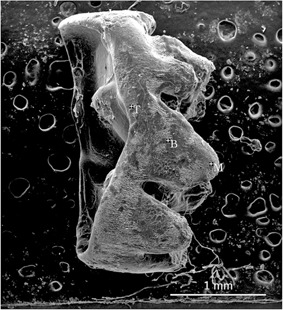
SEM/EDS examination of ${\text{Ca}}^{2+}/P^{3-}+{\text{Ca}}^{2+}$ at the cusp tip (T), at the edge of the root direction (B), and at the midpoint between the two (M).

As shown in Fig. 5, the degree of calcification progressively increased during the first 14 days of development. The typical formation of the enamel rod and dentin tubules was not observed in all samples (Fig. 5). Instead, we observed clump‐shaped calcification with pores.

**Figure 6:**
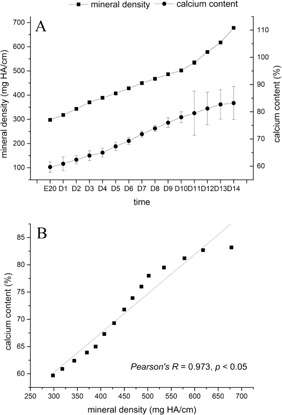
Mineral density (a) (measured by micro‐CT) and calcium content (measured by SEM) of dental hard tissue from E20 to D14. Pearson's correlation analysis (b), showing a strong correlation between mineral density measured by micro‐CT and calcium content measured by SEM.

Figure 6(a) shows the dental MD measured by micro‐CT and the ${\text{Ca}}^{2+}/P^{3-}+{\text{Ca}}^{2+}$ ratio (calcification) measured by SEM. Pearson's analysis shows a good linear correlation between mean MD and mean calcification (r=0.973, p<0.05), indicating that MD is strongly correlated with the ${\text{Ca}}^{2+}/P^{3-}+{\text{Ca}}^{2+}$ ratio (Fig. 6(b)). These results suggest that MD measured by micro‐CT is reliable.

## IV. DISCUSSION

Assessing dental MD by micro‐CT is a new way of measuring dental MD and, therefore, needed to be validated. We measured calcium and phosphorus levels using EDS, and the ${\text{Ca}}^{2+}/P^{3-}+{\text{Ca}}^{2+}$ ratio was used as a measure of calcification. In this present study, we used high‐resolution micro‐CT to obtain 3D images of the tooth germs of neonatal rats. Good visualization was achieved using a voxel size of 10 μm. The technique provided accurate visualization and integration of the anatomy and morphology of dental hard tissues. Using micro‐CT, we were able to show that dental hard tissue mineralization is an ongoing process, with hard tissue volume increasing in time, ultimately resulting in cusp formation. Furthermore, dental MD was strongly correlated with calcification, as suggested by previous studies.^14^, ^15^, ^16^, ^26^ This study suggests that micro‐CT is a promising method for monitoring tooth germ development, and that it can be used for basic and clinical dental research.

Micro‐CT systems were developed in the early 1980s to provide a miniaturized form of CT scanning with high spatial resolution with the range from 5 to 50 μm.^27^, ^28^ Micro‐CT has been increasingly used as a powerful technique for laboratory investigations. The method was further improved by advances in computer technologies, enabling the generation of thin‐section images of small specimens.^29^, ^30^

In the present study, micro‐CT provided a complete digital dataset that allowed us to assess the development of dental hard tissue, even if only minimal changes were observed from day to day. The technique allowed the nondestructive acquisition of the volume of interest in the form of a high‐resolution isotropic voxel volume, allowing the analysis of early dental hard tissue development and postnatal growth. In addition, tomographic reconstruction algorithms were used to provide 3D images of the tooth, allowing total stereoscopic visualization of its microarchitecture. Tomography also allows convenient extraction of appropriate sections from the 3D images, enabling micro‐CT to be used to more accurately analyze distinct regions of interest. Another interesting feature of micro‐CT is that it facilitates the creation of virtual cross sections of the sample in a number of orientations, meaning that the evaluation plane can be optimized for correct quantitative analysis. Furthermore, micro‐CT requires minimal sample manipulation. A new technique using synchrotron micro‐CT has recently been investigated and reported.^(31)^ However, it requires the treatment of samples with a silver‐based contrast agent, which was not necessary in the present study. In addition, that study only imaged mice teeth at E10, without measurements in time.

We recommend performing micro‐CT analysis prior to any histological processing of the samples, in order to prevent mechanical and physiochemical artifacts. Finally, the mean acquisition time using micro‐CT was approximately 6 s per section, which was considerably faster than the processing time using conventional histological methods. Thus, micro‐CT is an innovative tool with a number of advantages compared with the traditional histological approach.

A previous study showed in a passive smoking rat model that micro‐CT allowed the precise assessment of dental hard tissue microstructures and MD, while histological sections provided only the general structure with an undetermined error level.^(7)^ Since micro‐CT is an indirect method for assessing MD, we also used SEM/EDS, a quantitative method, that confirmed the results obtained using micro‐CT, as previously observed.^(7)^ Our results showed that the two variables are were closely correlated. A new technology, cone‐beam CT, has been shown to produce results that are similar to the ones obtained with micro‐CT.^(32)^ Two studies compared micro‐CT 3D reconstructions with physical measurements, and showed a difference of only 3%–5% between the two.^1^, ^33^ Thus, these studies suggest that 3D reconstruction of dental hard tissues using micro‐CT is accurate. A previous study used micro‐CT to assess rat tooth germ development, but the authors used only three time points (E20, D3, and D10), and could not provide a clear assessment of tooth germ development in time.^(7)^ The present study showed that micro‐CT is sensitive enough to assess dental hard tissue volume in rat embryos and neonates, showing a clear relation with time.

In previous studies, mineralization of hard tissues was traditionally measured using backscattered electron imaging under a SEM.^34^, ^35^ Indeed, this technique provides fine spatial resolution, and allows assessing remodeling activities, mechanical strength, and integrity. However, SEM is inherently invasive because of sample preparation and provides only two‐dimensional images. In our study, we used SEM to analyze changes in the atomic ratio of calcium atoms relative to the sum of calcium and phosphorus, and to confirm the changes measured using micro‐CT. Results showed that this ratio increased continually during hard tissue development, correlating with MD measured by micro‐CT. Accordingly, we showed that MD values measured by micro‐CT increased progressively during development of dental hard tissue, in the same way as mineralization measured by SEM. Therefore, micro‐CT could be deemed equivalent to SEM to assess the mineral content of mineralized tissues. In addition, micro‐CT could ultimately be performed in live animals, without the need for sample preparation and animal sacrifice.

## V. CONCLUSIONS

This preliminary study demonstrates that micro‐CT is an *ex vivo* nondestructive high‐resolution multiplanar and innovative tool for the evaluation of volume, MD, and degree of mineralization of dental hard tissue during development. Micro‐CT minimizes artifacts caused by sample preparation, and may be fully applicable in research on dental hard tissue development, as well as for integrated studies assessing the impact of dental fluorosis and tetracycline staining on mineralization.

## ACKNOWLEDGMENTS

This work was supported by the State Key Laboratory of Oral Disease (Sichuan University, China).
